# Genome-wide identification and abiotic stress response analysis of the *SUMO* family in alfalfa (*Medicago sativa* L.)

**DOI:** 10.7717/peerj.21276

**Published:** 2026-07-16

**Authors:** Ting Wang, Yupeng Guo, Yi Xu, Pengjun Shi, Xia Zheng

**Affiliations:** 1 Institute of Bast Fiber Crops, Chinese Academy of Agricultural Sciences, Changsha, China; 2 The College of Ecological Environment and Resources, Qinghai Minzu University, Xining, China

**Keywords:** Alfalfa, SUMO gene family, Gene family identification, Bioinformatics analysis, Expression analysis

## Abstract

SUMOylation is a well-conserved post-translational modification that is essential for modulating plant adaptation to various abiotic stresses. Although the functions of small ubiquitin-like modifier (SUMO) genes have been reported in various plant species, systematic studies focusing on the SUMO gene family members in alfalfa remain limited. In this study, we identified 49 MsSUMO genes from the alfalfa genome using bioinformatics approaches, and conducted comprehensive analyses of their phylogenetic relationships, structural features, cis-regulatory elements, and expression patterns. Most MsSUMO genes were predicted to localize in the nucleus and cytoplasm, consistent with their roles in transcriptional regulation and protein modification. Phylogenetic analysis grouped MsSUMO, soybean and Arabidopsis SUMO genes into seven subfamilies, which exhibited both high homology and species-specific divergence, suggesting functional differentiation during evolution. Conserved motif and domain analyses revealed strong structural consistency among MsSUMO members, with relatively simple gene architectures. In total, 59 types of cis-elements were detected in the promoter regions, playing crucial roles in plant growth, light signaling, and responses to biotic and abiotic stresses. Abscisic acid-responsive elements (ABREs) were the most abundant, implying that this gene family may serve key functions in stress regulation via the abscisic acid (ABA) signal pathway. Protein interaction network analysis indicated that MsSUMO members cooperate with core enzymes to modulate downstream stress-responsive targets. Transcriptome and real-time quantitative polymerase chain reaction (RT-qPCR) results showed that eight MsSUMO genes exhibited significant expression responses to salt, drought, and waterlogging stresses. Remarkably, six genes consistently exhibited upregulation across all three stress conditions. This observation underscores their potential as pivotal players in abiotic stress tolerance and identifies them as promising candidates for subsequent functional characterization.

## Introduction

Small ubiquitin-like modifier (SUMO) is generally considered a family of small proteins of approximately 100–115 amino acids in length, although the exact size varies among species and between family members. As a post-translational modification, SUMOylation regulates protein function in most eukaryotic cells by altering subcellular localization, protein–protein interactions, and protein stability (Castaño-Miquel et al., 2013). SUMOylation, also referred to as SUMO conjugation (Wei et al., 2018), is the biological process in which SUMO is covalently attached to lysine residues on target proteins through the sequential action of an E1 activating enzyme, an E2 conjugating enzyme, and an E3 ligase (Jing & Strader, 2023). Conversely, SUMO proteases can remove SUMO from substrates, indicating that SUMO modification is reversible. SUMO-specific proteases (SENPs) catalyze deSUMOylation by cleaving the isopeptide bond between SUMO and the substrate, thereby releasing SUMO to participate freely in subsequent SUMOylation cycles (Srivastava & Sadanandom, 2020). As a central regulatory mechanism of post-translational modification in biological processes, SUMOylation significantly influences substrate stability, subcellular localization, and protein–protein interactions. Structurally, SUMO molecules exhibit a protein fold similar to that of ubiquitin (Johnson, 2004), and their conjugation mechanisms are also comparable (Han et al., 2018). Specifically, SUMO proteins belong to the *Ubi1_cv_Nsp3_Nlike* superfamily (accession: cl28922), which is characterized by a conserved ubiquitin-like (UBL) fold architecture. This structural hallmark distinguishes SUMO members from other modifiers and is essential for their recognition by SUMO-specific enzymatic machinery.

SUMO modification plays critical roles in numerous biological processes, including transcription, cell cycle progression, DNA damage responses, and signal transduction (Schwienhorst, Johnson & Dohmen, 2000). Studies have shown that this modification system participates in protein transport, transcription, translation, and signaling in yeast, while also facilitating stress responses under various environmental conditions (Seeler & Dejean, 2003). In animals, SUMOylation regulates early embryonic development and pathological processes (Nacerddine et al., 2005; Xu et al., 2020). In plants, SUMOylation is widely involved in growth, development, flowering, stress responses, and pathogen defense, constituting an indispensable regulatory mechanism throughout the plant life cycle (Sang & Wang, 2023). Genes of the SUMO family drive the synthesis of SUMO proteins, providing substrates for subsequent SUMOylation reactions. Through coordinated actions with SUMO-conjugating enzymes (SCE), E3 ligases, and deSUMOylating enzymes, they collectively regulate the modification levels of target proteins, thereby playing essential roles in plant stress tolerance and immunity. In maize, overexpression of the SUMO-conjugating enzyme gene *ZmSCE1b* significantly enhances plant tolerance to paraquat (Wang et al., 2021). Studies in soybean have revealed stress-specific response patterns: under heat stress, *GmSCEd* and *GmE3f* accumulate in roots and stems, whereas during *Phytophthora* infection, *GmSUMO2/3* are primarily activated in roots. These findings indicate that SUMO modification confers dual resistance to both biotic and abiotic stresses in soybean through spatiotemporal regulatory mechanisms (Li et al., 2019). The *OsSCE* gene family in rice exhibits functional diversity, with *OsSCE3* specifically involved in drought stress responses, while *OsSCE1* primarily regulates carbohydrate metabolism (Joo et al., 2019). Moreover, the expression levels of SUMO-binding proteins in soybean are differentially regulated by salt, heat, and abscisic acid (ABA) signaling, further confirming the sensitivity of the SUMO modification system to environmental cues (Li et al., 2017). Research in the model plant *Arabidopsis thaliana* has provided key insights into the immune regulatory functions of SUMO. Disruption of the interaction between SUMO E1 and E2 enzymes suppresses SUMOylation *in vivo* and significantly reduces plant defense responses against necrotrophic fungal pathogens, highlighting the positive regulatory role of SUMO proteins in immune responses (Castaño-Miquel et al., 2017). Meanwhile, members of the *Arabidopsis* SUMO gene family (*SUMO1*–*SUMO8*) display target specificity during stress responses, with individual SUMO isoforms participating in diverse stress responses through recognition of distinct substrates (Kurepa et al., 2003).

Alfalfa (*Medicago sativa* L.) is a perennial forage legume that thrives in warm and semi-arid climates. Owing to its strong adaptability, high yield, and superior nutritional quality, it has become one of the most widely cultivated and economically valuable forage legumes worldwide (Qi et al., 2008). However, the continuous expansion of alfalfa cultivation has increased its exposure to various abiotic stresses during growth, which adversely affects normal development and limits large-scale production (Quan, Yu & Liu, 2012). As an autotetraploid species (2*n* = 4*x* = 32), alfalfa exhibits high sequence homology among gene family members, which may drive functional diversification within the SUMO gene family and thereby enhance its adaptability to diverse environmental conditions. Nevertheless, the systematic identification and functional differentiation of the *MsSUMO* gene family, particularly under multiple abiotic stresses, remain largely unexplored. Given the limited information available regarding the roles of this gene family in abiotic stress responses, this study identified members of the SUMO gene family in alfalfa and performed comprehensive computational analyses to investigate their differential expression under salt, drought, and waterlogging stresses. These findings provide a theoretical basis for future functional studies and offer new molecular insights for stress tolerance breeding in alfalfa.

## Materials and Methods

### Materials

Seeds of the alfalfa cultivar “Gongnong No.1” were selected for uniformity and sown into pots (12 cm diameter × 12 cm height). The growth substrate consisted of nutrient soil and vermiculite mixed at a 2:1 (v/v) ratio. Five seeds were sown per pot at a depth of one cm. Pots were grown in a controlled-environment growth chamber under the following conditions: 23 °C (day)/18 °C (night), a 16 h light/8 h dark photoperiod, photosynthetic photon flux density of 245 µmol m^−2^s^−1^, and relative humidity of 60–70%. Leaves from 3-week-old seedlings grown under these conditions were collected for expression analysis under abiotic stresses. Prior to stress treatments, 3-week-old plants with uniform growth were selected. Stress treatments were applied as follows: waterlogging stress was imposed using a double-pot method with the water level maintained two cm above the soil surface; Salt stress was simulated by irrigating plants with 200 mmol L^−1^ NaCl solution every two days, while drought stress was induced by irrigation with 15% (w/v) PEG-6000 solution every two days. Leaf samples were collected at 0, 3, 6, and 12 h after treatment, immediately snap-frozen in liquid nitrogen, and stored at −80 °C until further analysis. All treatments included three biological replicates.

### Methods

#### Identification of the *MsSUMO* gene family in alfalfa

Identification of the MsSUMO gene family was performed using HMM-based structural screening. Initially, the HMMER 3.0 program was employed to conduct a genome-wide scan of the alfalfa proteome using the SUMO domain (PF13881) to identify MsSUMO genes. The resulting sequences were further validated with the NCBI Batch CD Search tool to confirm the presence of the conserved ubiquitin-like fold characteristic of SUMO.

#### Physicochemical property analysis of *MsSUMO* proteins

The physicochemical properties of *MsSUMO* proteins, including their amino acid composition, molecular weight, isoelectric point, and grand average of hydropathicity, were predicted with the ProtParam tool available on the ExPASy website (https://web.expasy.org/protparam/). In addition, the subcellular localization of the family members was predicted using WoLF PSORT (https://wolfpsort.hgc.jp/).

#### Phylogenetic analysis of the *MsSUMO* gene family

To investigate the evolutionary relationships of the SUMO gene family, protein sequences from alfalfa, *Arabidopsis* and soybean were collected. Multiple sequence alignment was performed using the MUSCLE program with default parameters. A phylogenetic tree was constructed using the neighbor-joining method implemented in MEGA 11 (Tamura, Stecher & Kumar, 2021), with 1,000 bootstrap replicates. The resulting tree was visualized and annotated using iTOL. To enhance statistical rigor, only bootstrap values ≥50 were displayed at the nodes.

#### Analysis of gene structure, conserved motifs, and cis-acting elements of the *MsSUMO* gene family in alfalfa

Conserved motifs within the *MsSUMO* gene family were identified using the MEME online tool (http://meme.sdsc.edu/meme/cgi-bin/meme.cgi). The maximum number of motifs was set to 25 to ensure exhaustive identification of conserved sequences. Subsequently, the top 10 motifs that were statistically significant (*E*-value) and consistently distributed among family members were selected for final visualization and analysis. The NCBI Conserved Domain Database (CDD) (https://www.ncbi.nlm.nih.gov/Structure/cdd/wrpsb.cgi) was utilized for protein domain analysis, with subsequent visualization of the findings carried out using TBtools software (Chen et al., 2023). Promoter sequences (1,500 bp upstream of the start codon) of the MsSUMO genes were extracted using the FASTA Extractor tool in TBtools, with the “Up Stream Bases” parameter set to 1,500 and “Retain Only Upstream or Downstream Bases” selected. The extracted sequences were submitted to the PlantCARE database for cis-acting regulatory element prediction. The types and numbers of cis-acting elements present in each gene promoter were calculated and visualized accordingly.

#### Chromosomal localization and collinearity analysis of the *MsSUMO* gene family in alfalfa

Chromosomal positions of *MsSUMO* genes were obtained from the genome annotation data of alfalfa. Gene density files were generated using the Gene Density Profile tool in TBtools, and gene positions were visualized using the Gene Location Visualize (Advanced) function. Collinearity among *MsSUMO* genes was analyzed and visualized using the One Step MCScanX module. Chromosome IDs and lengths of alfalfa were obtained using the FASTA Stats tool. The One Step MCScanX-Super Fast plugin was used to identify collinear gene pairs within the *MsSUMO* gene family, and the results were visualized with the Advanced Circos tool. Intra-species collinearity within alfalfa and inter-species collinearity among *MsSUMO*, *AtSUMO*, and *GmSUMO* genes were also detected using the One Step MCScanX module in TBtools and visualized accordingly.

#### Protein-protein interaction network prediction

The protein–protein interaction (PPI) network was predicted using the STRING database (https://string-db.org/). Given the high sequence homology between *Medicago sativa* and *Medicago truncatula*, the SUMO protein sequences of *Medicago sativa* were aligned to their corresponding homologous proteins in *Medicago truncatula* to construct the protein interaction network. The predicted interaction network was subsequently visualized using Cytoscape software.

#### Expression pattern analysis of the *MsSUMO* gene family in Alfalfa

Transcriptome data of the *MsSUMO* gene family under salt, drought, and waterlogging stress conditions in alfalfa were downloaded from the public database AlfalfaGEDB (http://alfalfagedb.liu-lab.com). The expression levels of *MsSUMO* genes under salt, drought, and waterlogging stresses in this database.

#### RT-qPCR analysis of *MsSUMO* genes

To determine whether the *MsSUMO* gene family responds to abiotic stress signals, total RNA was extracted from the leaves of treated alfalfa “Gongnong No. 1” plants using the SteadyPure Plant RNA Extraction Kit (Accurate Biotechnology, Hunan, China) following the manufacturer’s instructions. The concentration and purity of the extracted RNA were assessed using a NanoDrop 2000 spectrophotometer (Thermo Fisher Scientific, Waltham, MA, USA), and RNA integrity was verified by 1% agarose gel electrophoresis. RNA was reverse-transcribed to cDNA using the Evo M-MLV RT Mix Kit with gDNA Clean for qPCR Ver.2 (Accurate Biotechnology, Hunan, China) to remove residual genomic DNA and synthesize cDNA, and quantitative real-time PCR (RT-qPCR) was performed using the CFX96 Real-Time PCR Detection System (Bio-Rad Laboratories, Hercules, CA, USA). The primer sequences are listed in Table 1. The 20 µL reaction mixture contained: 10 µL 2× SYBR Green Pro Taq HS Premix (Accurate Biotechnology, Hunan, China), 0.4 µL of forward and reverse primers (10 µmol/L each), 2 µL cDNA template, and 7.2 µL RNase-free water. Primers were designed using the Primer 3.0 tool (https://primer3.ut.ee/). Reference genes were selected under different stress conditions (Xu et al., 2026): *UBL-2a* for salt stress, *Rer1* for drought stress, and *ADF* for waterlogging stress. The PCR program was as follows: pre-denaturation at 95 °C for 30 s; 40 cycles of denaturation at 95 °C for 10 s, annealing at 56 °C for 30 s; followed by a melt curve analysis from 60 °C to 95 °C. Primers specificity was confirmed by melt curve analysis showing a single peak for each assay. Relative gene expression levels were calculated using the 2^−ΔΔCT^ method (Chou & Shen, 2010).

**Table 1:** The primer sequences of RT-qPCR.

**Gene symbol**	**Primers sequence (5′–3′)**	**Product size (bp)**
*UBL-2a*	F: CCAAACCCAAACTCACCAG	108
	R: AGCAGTCCAACTCTGCTCAAC	
*Rer1*	F: GCCTTCTGATGGTGGACCT	165
	R: GGCCAGAAGACAGGAACATC	
*ADF*	F: GCATCTGGTATGGCAGTCC	183
	R: GCACTCATCAGCAGGAAGG	
*MS.gene30916.t1*	F: CCTGACCAACAGCGTCTCAT	97
	R: CAAGGTGAAGCGTGGACTCT	
*MS.gene006985.t1*	F: CTACAACTGTGGGTGCCCTT	83
	R: ATCCACAAACACTCCCCACC	
*MS.gene071146.t1*	F: ATTCCCCCAGACCAGCAAAG	125
	R: ATGCCTCCCCTCAAACGAAG	
*MS.gene063025.t1*	F: ACGTCAAGGCCAAGATCCAG	121
	R: AAACCCTAGCAGACGCGAAA	
*MS.gene97484.t1*	F: CTTCGTTTGAGGGGAGGCAT	142
	R: TCTGCTGATCTGGTGGGATT	
*MS.gene86087.t1*	F: TGGACACGACATTGACACGT	116
	R: TGATTGAAGGCGTGTCCTGG	
*MS.gene031139.t1*	F: CCAGACACGACATTGACACG	86
	R: TACACGGCACCGACAATTGT	
*MS.gene045431.t1*	F: CTTCGTGGTGGCATGCAAAT	173
	R: CGACCATCCTCCAACTGCTT	

## Results

### Identification of the *MsSUMO* gene family members in alfalfa

Based on the results of the hmmsearch program and further screening using the Batch CD-Search tool on the NCBI website, sequences lacking the typical SUMO domain were excluded. A total of 49 *MsSUMO* genes were identified from the alfalfa genome. The encoded protein sequences range from 104 amino acids (*MS.gene006985.t1*) to 381 amino acids (*MS.gene39507.t1*), with predicted molecular weights ranging from 11,289 Da (*MS.gene006985.t1*) to 42,714.7 Da (*MS.gene39507.t1*). The theoretical points isoelectric (pI) of the proteins vary from 4.98 (*MS.gene84094.t1*) to 10.32 (*MS.gene73596.t1*). Subcellular localization predictions indicated that 45 MsSUMO proteins were localized in the cytoplasm and 45 in the nucleus, with 29 predicted to be localized in both compartments (Table 2). This localization pattern is consistent with the functional roles of SUMO proteins in nucleo-cytoplasmic transport and transcriptional regulation.

**Table 2:** *Medicago* *sativa* *SUMO* gene family information.

**Gene ID**	**Amino acid length (aa)**	**Molecular weight (Da)**	**Isoelectric point (pI)**	**Chromosomal location**	**Predicted subcellular localization^1^**
*MS.gene000531.t1*	150	16,679.9	6.00	chr4.3	Cyto; Nucl; Chlo; Extr; E.R.
*MS.gene001903.t1*	254	29,162.0	5.17	chr7.3	Cyto; Mito; Nucl; Chlo; Plas; Extr
*MS.gene001904.t1*	152	17,032.6	8.69	chr7.3	Cyto; Cysk; Nucl; Extr; Plas; Golg
*MS.gene006731.t1*	305	34,192.0	7.82	chr4.1	Cyto; Cyto_Nucl; Chlo; Pero
*MS.gene006733.t1*	305	34,208.0	7.81	chr4.1	Cyto; Cyto_Nucl; Chlo; Pero
*MS.gene006734.t1*	305	34,208.0	7.81	chr4.1	Cyto; Cyto_Nucl; Chlo; Pero
*MS.gene006739.t1*	305	34,238.0	7.81	chr4.1	Cyto; Cyto_Nucl; Chlo; Pero
*MS.gene006742.t1*	155	17,201.7	6.27	chr4.1	Cyto_Nucl; Cyto; Nucl; Mito; Chlo; E.R.
*MS.gene017181.t1*	153	17,094.5	5.85	chr5.3	Cyto; Cyto_Nucl; Chlo; Mito; Plas
*MS.gene021818.t1*	119	12,879.6	7.95	chr7.1	Nucl; Chlo; Cyto; Extr; Mito
*MS.gene022937.t1*	229	25,685.3	7.76	chr7.4	Cyto; Cyto_Nucl; Chlo; Mito; Pero
*MS.gene030472.t1*	116	12,826.7	8.51	chr4.4	Nucl; Cyto; Chlo; Extr
*MS.gene031139.t1*	117	12,692.7	7.81	chr6.2	Chlo; Extr
*MS.gene031566.t1*	155	17,201.7	6.27	chr4.4	Cyto_Nucl; Cyto; Nucl; Mito; Chlo; E.R.
*MS.gene038416.t1*	153	17,094.5	5.85	chr5.4	Cyto; Cyto_Nucl; Chlo; Mito; Plas
*MS.gene040727.t1*	231	25,795.4	6.82	chr1.2	Cyto; Cyto_Nucl; Chlo; Mito
*MS.gene044829.t1*	301	34,644.2	5.24	chr7.2	Nucl; Cyto; Cysk; Plas
*MS.gene045423.t1*	155	17,201.7	6.27	chr4.2	Cyto_Nucl; Cyto; Nucl; Mito; Chlo; E.R.
*MS.gene045426.t1*	305	34,238.0	7.81	chr4.2	Cyto; Cyto_Nucl; Chlo; Pero
*MS.gene045431.t1*	305	34,208.0	7.81	chr4.2	Cyto; Cyto_Nucl; Chlo; Pero
*MS.gene045433.t1*	305	34,208.0	7.81	chr4.2	Cyto; Cyto_Nucl; Chlo; Pero
*MS.gene045436.t1*	305	34,192.0	7.82	chr4.2	Cyto; Cyto_Nucl; Chlo; Pero
*MS.gene057527.t1*	137	14,904.0	6.91	chr7.2	Cyto; Chlo; Nucl; Extr; Cysk
*MS.gene062293.t1*	153	17,094.5	5.85	chr5.1	Cyto; Cyto_Nucl; Chlo; Mito; Plas
*MS.gene063025.t1*	224	25,350.9	6.37	chr4.2	Cyto; Nucl; Extr
*MS.gene063276.t1*	138	15,137.2	9.68	chr4.2	Cyto; Nucl; Chlo
*MS.gene065503.t1*	153	17,094.5	5.85	chr5.2	Cyto; Cyto_Nucl; Chlo; Mito; Plas
*MS.gene006985.t1*	381	11,289.0	8.99	chr4.1	Cyto; Nucl; Chlo
*MS.gene070758.t1*	305	34,192.0	7.82	chr4.2	Cyto; Cyto_Nucl; Chlo; Pero
*MS.gene070760.t1*	305	34,192.0	7.82	chr4.2	Cyto; Cyto_Nucl; Chlo; Pero
*MS.gene070761.t1*	305	34,192.0	7.82	chr4.2	Cyto; Cyto_Nucl; Chlo; Pero
*MS.gene071145.t1*	229	25,685.3	7.76	chr7.4	Cyto; Cyto_Nucl; Chlo; Mito; Pero
*MS.gene071146.t1*	229	25,685.3	7.76	chr7.2	Cyto; Cyto_Nucl; Chlo; Mito; Pero
*MS.gene073704.t1*	231	25,847.4	6.98	chr1.1	Cyto; Cyto_Nucl; Chlo; Plas; E.R.; Pero
*MS.gene073771.t1*	231	25,809.4	6.82	chr1.1	Cyto; Cyto_Nucl; Chlo; Mito
*MS.gene23010.t1*	129	14,043.2	9.05	chr7.2	Cyto; Nucl; Chlo; Cysk
*MS.gene24581.t1*	231	25,809.4	6.82	chr1.3	Cyto; Cyto_Nucl; Chlo; Mito
*MS.gene30916.t1*	231	25,809.4	6.82	chr1.2	Cyto; Cyto_Nucl; Chlo; Mito
*MS.gene39507.t1*	381	42,714.7	7.85	chr4.2	Cyto; Cyto_Nucl; Chlo; Plas; Pero
*MS.gene45247.t1*	137	14,918.0	6.91	chr7.3	Chlo; Nucl; Cyto; Cysk; Extr
*MS.gene73595.t1*	298	34,786.5	6.27	chr7.1	Nucl; Cyto; Chlo; Plas; Cysk
*MS.gene73596.t1*	137	15,128.4	10.32	chr7.1	Nucl; Cyto; Extr; Chlo; Cysk; Golg
*MS.gene79465.t1*	116	12,836.7	8.51	chr4.1	Cyto; Nucl; Chlo; Extr
*MS.gene84094.t1*	192	21,454.2	4.98	chr1.4	Cyto; Cyto_Nucl; Chlo; Mito; Plas
*MS.gene86087.t1*	117	12,692.7	7.81	chr6.3	Chlo; Extr
*MS.gene86688.t1*	254	29,267.1	5.36	chr7.4	Mito; Cyto; Chlo; Nucl; Plas; Pero
*MS.gene97484.t1*	229	25,685.3	7.76	chr7.4	Cyto; Cyto_Nucl; Chlo; Mito; Pero
*MS.gene97486.t1*	229	25,685.3	7.76	chr7.3	Cyto; Cyto_Nucl; Chlo; Mito; Pero
*MS.gene97583.t1*	137	14,918.0	6.91	chr7.4	Chlo; Nucl; Cyto; Cysk; Extr

**Notes.**

1 Subcellular localization was predicted using the WoLF PSORT online tool.

Abbreviations Cyto cytoplasm Nucl nucleus Cyto Nucl dual localization in both cytoplasm and nucleus Chlo chloroplast Mito mitochondria Plas plasma membrane E.R. endoplasmic reticulum

### Phylogenetic analysis of the *MsSUMO* gene family in alfalfa

**Figure 1:**
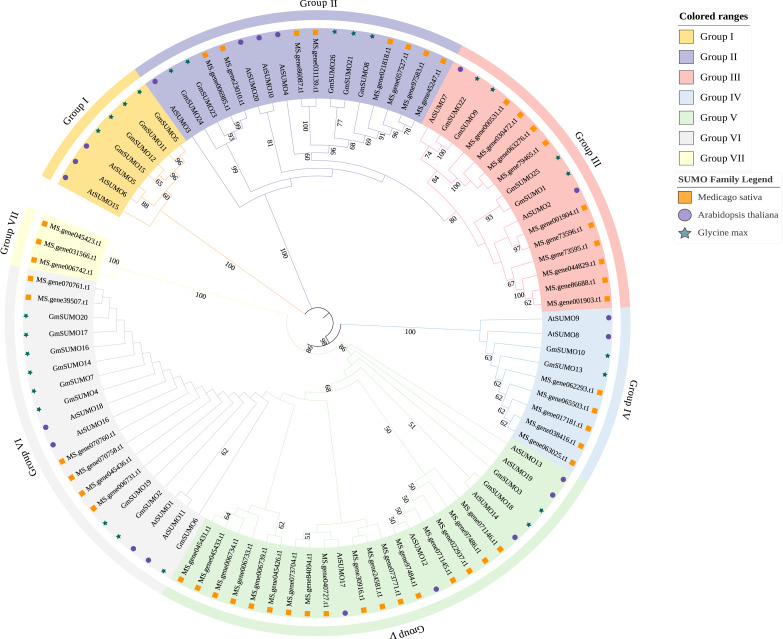
Phylogenetic tree of *SUMO* gene family from *Medicago sativa*, *Arabidopsis thaliana* and *Glycine max*. A total of 95 SUMO protein sequences (49 from *Medicago sativa*, 20 from *Arabidopsis thaliana*, and 26 from *Glycine max*) were aligned using MUSCLE. The 95 SUMO members are clustered into seven distinct subfamilies (Group I–VII), indicated by different colored arcs. Shapes represent different species.

To investigate the evolutionary relationships of the SUMO gene family in alfalfa, a phylogenetic tree was constructed based on 95 SUMO protein sequences, including 49 *MsSUMO*, 20 *AtSUMO*, and 26 *GmSUMO* members (Fig. 1). Based on the phylogenetic relationships, the SUMO genes were classified into seven groups. Group V and Group III were the largest clades, containing 17 and 10 *MsSUMO* members, respectively. *MsSUMO* and *GmSUMO* exhibited closer clustering patterns. For example, in Group II and Group IV, SUMO proteins from alfalfa and soybean formed more compact branches, consistent with the evolutionary relationship among leguminous species. In contrast, Group VII consisted exclusively of *MsSUMO* members, suggesting potential lineage-specific expansion during evolution. However, the terminal branches of Group VI exhibited relatively low bootstrap support, which may be associated with the high sequence conservation among genome members in autotetraploid alfalfa.

### Analysis of conserved motifs, gene structure, and Cis-acting elements of the *MsSUMO* gene family in alfalfa

**Figure 2:**
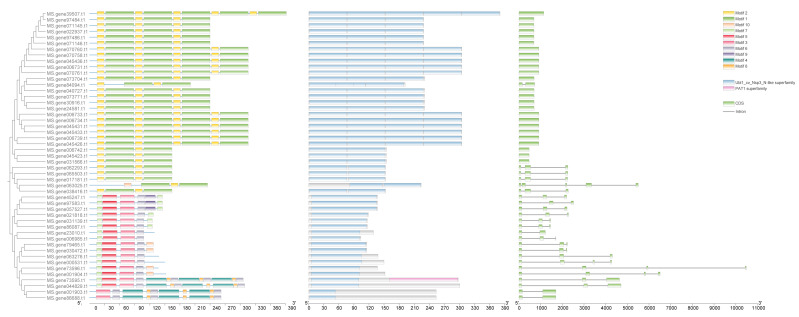
Conserved motif, gene structure, and domain composition of the *MsSUMO* gene family in alfalfa. (A) Distribution of conserved motifs predicted by MEME (max motifs = 25). (B) Gene structures including UTRs, CDS, and introns were visualized using TBtools. (C) Conserved domains were identified using NCBI CDD and grouped into two main types. Colored boxes represent different motifs or domains.

**Figure 3:**
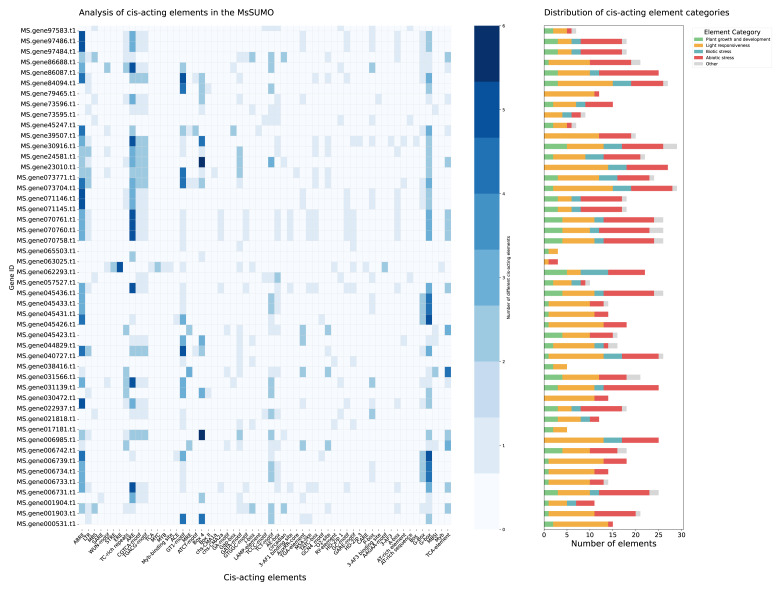
Cis-acting regulatory elements in the promoter regions (1,500 bp upstream) of *MsSUMO* genes. Elements were categorized into five functional groups: light responsiveness, growth and development, biotic stress, abiotic stress, and hormone responsiveness. Prediction was performed using PlantCARE.

To explore the structural characteristics of the *MsSUMO* gene family in alfalfa, a comprehensive visualization was conducted by integrating conserved motifs, untranslated regions (UTRs), coding sequences (CDS), introns, and conserved domains (Fig. 2). Using the online MEME tool, a total of 10 conserved motifs were predicted among the 49 *MsSUMO* genes (Fig. 2A). A total of 31 *MsSUMO* family members contain both Motif 1 and Motif 2, indicating that these two motifs are essential for the functional domain activity. The remaining 18 members possess more than four conserved motifs, predominantly Motif 3 and Motif 6. Additionally, four members contain both Motif 4 and Motif 8. Proteins such as *MS.gene073704.t1*, *MS.gene073771.t1*, and *MS.gene040727.t1* shared similar motif patterns with Motif 1 and Motif 2, while *MS.gene79465.t1*, *MS.gene063276.t1*, and *MS.gene000531.t1* exhibited common Motif 3, Motif 5, Motif 6, and Motif 7 arrangements. These similarities in motif composition also corresponded to closer phylogenetic relationships. In contrast, *MS.gene057527.t1*, *MS.gene45247.t1*, and *MS.gene97583.t1* were more distantly related to other *MsSUMO* genes, which may be attributed to the presence of a unique and continuous Motif 9, possibly conferring specialized functions. The analysis of conserved domains among *MsSUMO* family members revealed that they could be divided into two subgroups based on their domain composition (Fig. 2C). One subgroup contained both the Ubi1_cv_Nsp3_N-like superfamily domain and the PAT1 superfamily domain, while the other subgroup harbored only the Ubi1_cv_Nsp3_N-like superfamily domain. These findings point to the functional conservation of the alfalfa *MsSUMO* gene family, suggesting its classification as a unified functional family. In this study, to further predict the potential functions of the *MsSUMO* gene family in alfalfa, we analyzed the promoter regions of the *MsSUMO* genes. The 1,500 bp upstream sequences of the *MsSUMO* genes were retrieved and subjected to cis-acting element prediction using the PlantCARE database (Fig. 3). A total of 59 cis-acting elements were identified in the promoter regions of *MsSUMO* genes, which were classified into five categories: plant growth and development, light responsiveness, biotic stress, abiotic stress, and others. Elements associated with plant growth and development included MSA-like, HD-Zip, and O2-site. Light-responsive elements included AE-box, GT1-motif, and Box 4. Biotic stress-related elements included WUN-motif, SARE, and LTR, whereas ABRE, HSE, and ARE were associated with abiotic stress. Among these, ABRE elements were the most frequently detected in the *MsSUMO* promoters. Moreover, nearly all *MsSUMO* genes contained at least one hormone-responsive element in their promoter regions.

### Chromosomal localization and gene collinearity analysis

**Figure 4:**
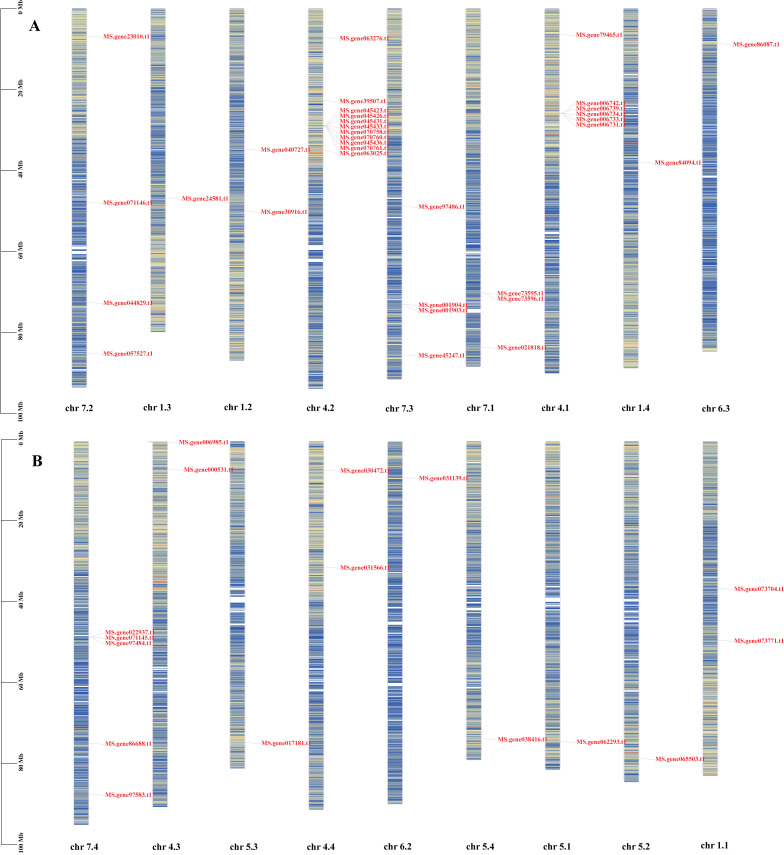
Chromosomal localization of *MsSUMO* genes in alfalfa. A total of 49 *MsSUMO* genes were mapped on 18 chromosomes using the Gene Location Visualize (Advanced) tool in TBtools.

**Figure 5:**
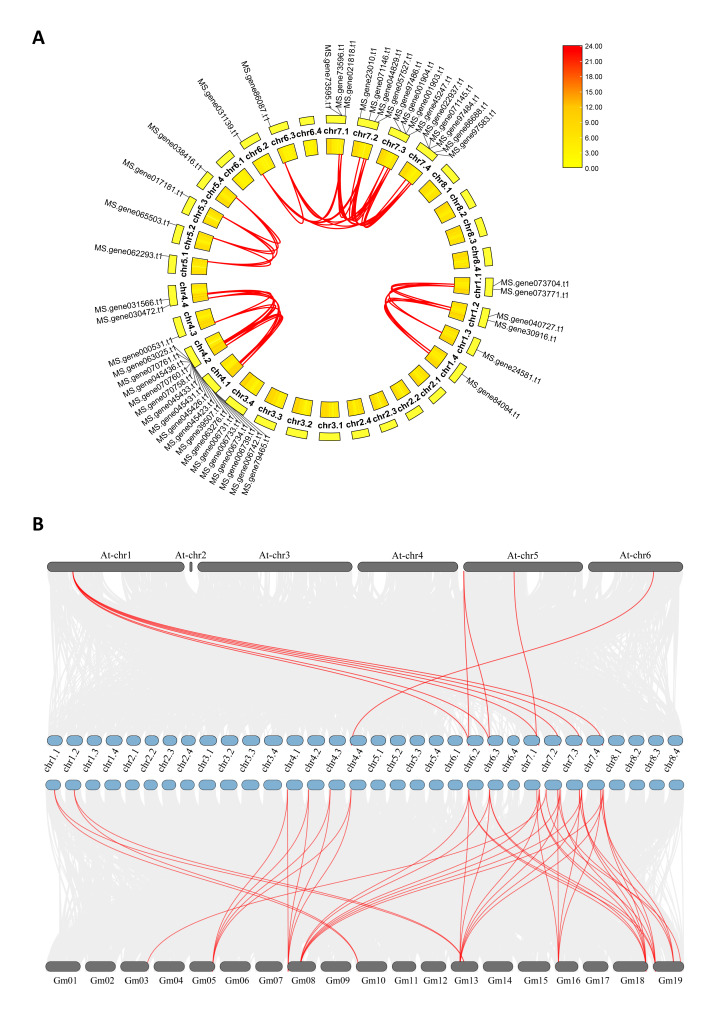
Collinearity analysis of the *MsSUMO* gene family. (A) Intra-species collinearity within alfalfa shows multiple duplicated gene pairs. (B) Inter-species collinearity among alfalfa, Arabidopsis, and soybean were visualized using TBtools. Segmentally duplicated gene pairs are connected by colored lines.

In this study, the chromosomal distribution of the *MsSUMO* gene family in alfalfa was analyzed (Fig. 4). The results showed that the 49 *MsSUMO* genes were unevenly distributed across 18 chromosomes. The highest number of genes was observed on chr 4.2, which harbored 11 *MsSUMO* genes. Chr 4.1 contained six genes, chr 7.4 contained five genes, chr 7.2 and 7.3 each carried four genes, and chr 7.1 had three genes. Chr 1.1, 1.2, and 4.4 each carried two *MsSUMO* genes, while the remaining chromosomes (chr1.3, chr1.4, chr4.3, chr5.1, chr5.2, chr5.3, chr5.4, chr6.2, and chr6.3) each contained only one gene. Notably, several genes, including *MS.gene23010.t1*, *MS.gene063276.t1*, *MS.gene79465.t1*, *MS.gene86087.t1*, *MS.gene000531.t1*, *MS.gene030472.t1*, and *MS.gene031139.t1*, were located near the telomeric regions of chromosomes. In contrast, *MS.gene006742.t1* and *MS.gene045423.t1* were clustered together, and genes such as *MS.gene057527.t1* and *MS.gene45247.t1* were positioned at the distal ends of the chromosomes. In the collinearity analysis of *MsSUMO* genes in alfalfa, we found that most *MsSUMO* family members were located on different chromosomes yet exhibited extensive collinear relationships (Fig. 5A). In addition, several segmental duplication events were identified between adjacent chromosomes (Hammoudi et al., 2016). For example, *MS.gene073704.t1* and *MS.gene073771.t1* located on homologous chr1.1 showed segmental duplication with genes on chr1.2 and chr1.4. Similarly, *MS.gene30916.t1* on chr1.2 and *MS.gene24581.t1* on chr1.3 also shared a segmental duplication relationship. To further investigate the evolutionary mechanisms and phylogenetic relationships of the *MsSUMO* gene family, a comparative collinearity analysis was conducted among alfalfa, arabidopsis, and soybean (Fig. 5B). The results revealed the presence of collinear relationships among the three species. Several *MsSUMO* genes exhibited significant orthologous relationships with *AtSUMO* genes, particularly those located on At-chr1, At-chr5, and At-chr6, suggesting that SUMO genes are fundamentally conserved across different dicotyledonous species. Moreover, a higher degree of collinearity was observed between alfalfa and soybean. Most *MsSUMO* genes exhibited one-to-many collinear relationships with *GmSUMO* chromosomes (such as Gm08, Gm13, and Gm19), indicating a closer evolutionary relationship between alfalfa and soybean as leguminous species. Compared with Arabidopsis, alfalfa and soybean share stronger orthologous relationships, suggesting that SUMO genes are highly conserved during legume evolution and may play important roles in species-specific development and stress adaptation.

### Prediction of *MsSUMO* protein-protein interaction network and functional targets

**Figure 6:**
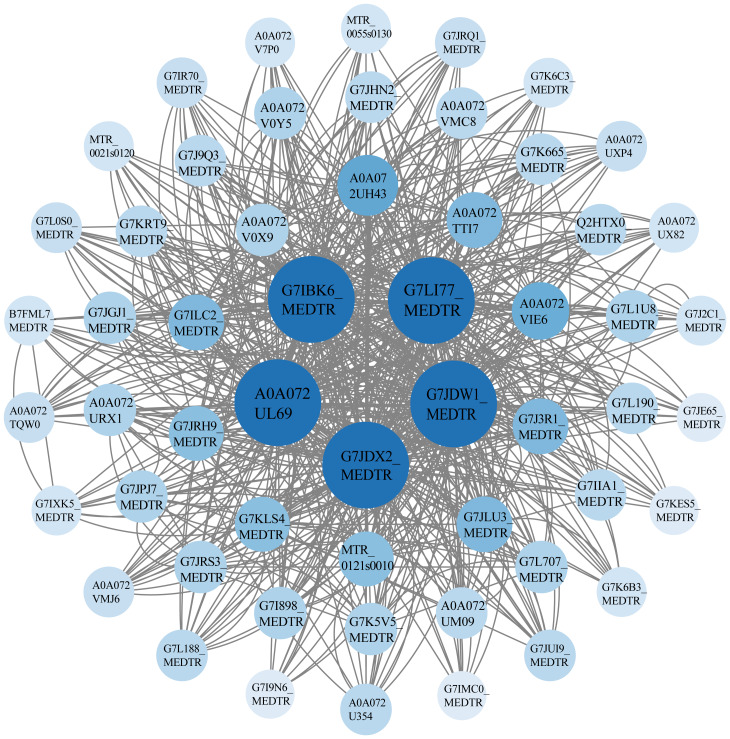
Predicted protein-protein interaction network of the 49 *MsSUMO* proteins. The network was constructed based on orthologs in *Medicago truncatula*. Central hub nodes, including G7IBK6 (SUMO-conjugating enzyme E2), G7LI77 (SUMO E3 ligase), and A0A072 UL69 (SUMO-specific protease), represent the core components of the SUMOylation machinery. These hubs exhibit extensive interactions with various stress-responsive proteins, suggesting a complex regulatory landscape for abiotic stress adaptation in alfalfa.

To further investigate the potential regulatory mechanisms and biological relevance of the *MsSUMO* gene family in alfalfa, PPI network was constructed (Fig. 6). The regulatory network of the *MsSUMO* family includes G7IBK6 (SUMO-conjugating enzyme E2) and G7LI77 (SUMO E3 ligase), which exhibit extensive interactions with multiple stress-responsive proteins. This network architecture suggests that *MsSUMO* family members function cooperatively with these core enzymes to regulate downstream targets in response to abiotic stress.

### Expression profiling of *MsSUMO* genes under abiotic stress in alfalfa

**Figure 7:**
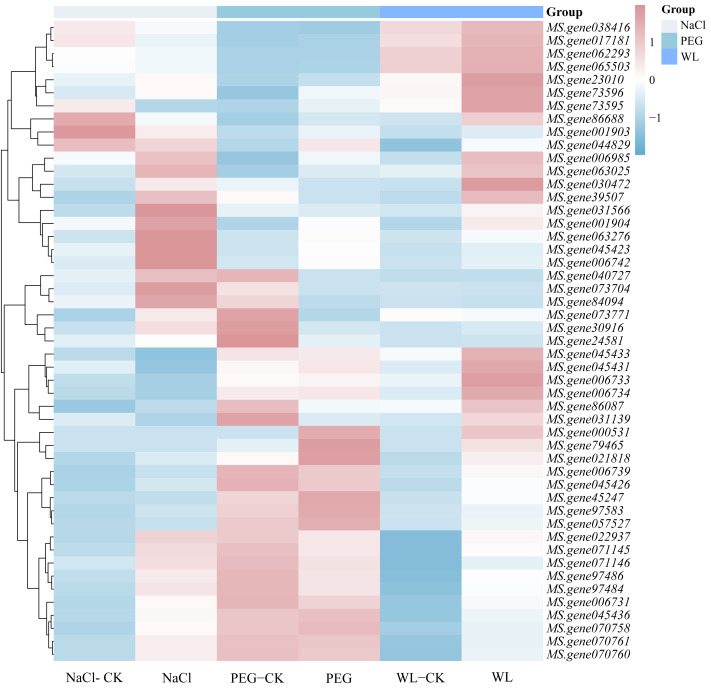
Expression heatmap of 49 *MsSUMO* genes under salt (NaCl), drought (PEG), and waterlogging (WL) stress conditions based on transcriptome data (FPKM values log_2_(FPKM + 1)). Data were clustered by hierarchical clustering using TBtools. Red indicates high expression; blue indicates low expression. CK stands for Control, representing the specific non-treated samples associated with each respective stress dataset.

To further explore the response of alfalfa to abiotic stress, transcriptome data under three stress conditions (salt (NaCl), drought (PEG), and waterlogging (WL)) were downloaded from the AlfalfaGEDB database. The expression levels of 49 *MsSUMO* genes were normalized using FPKM values and transformed by log_2_(FPKM + 1). A hierarchical clustering analysis was performed, and a heatmap was generated to visualize expression differences among the *MsSUMO* family members (Fig. 7). The results indicated that different *MsSUMO* genes exhibited diverse expression patterns in response to different abiotic stresses. Under salt stress (NaCl), genes such as *MS.gene038416*, *MS.gene006985*, *MS.gene063025*, *MS.gene30916*, *MS.gene030472*, *MS.gene073704*, and *MS.gene045431* were significantly upregulated compared to the control, while *MS.gene045431* and *MS.gene031139* were downregulated. Under drought stress (PEG), genes including *MS.gene073771*, *MS.gene24581*, *MS.gene86087*, and *MS.gene031139* showed decreased expression, whereas *MS.gene044829*, *MS.gene79425*, and *MS.gene045431* were positively regulated. In response to waterlogging stress (WL), most genes—such as *MS.gene23010*, *MS.gene045431*, *MS.gene86087*, and *MS.gene031139*—displayed upregulated expression. Interestingly, several genes including *MS.gene73596*, *MS.gene006985*, *MS.gene071146*, *MS.gene063025*, *MS.gene97484*, *MS.gene86087*, *MS.gene031139*, and *MS.gene045431* were co-induced under all three stress conditions, suggesting their potential roles as core regulatory genes involved in multi-stress response mechanisms.

### RT-qPCR analysis of MsSUMO genes

**Figure 8:**
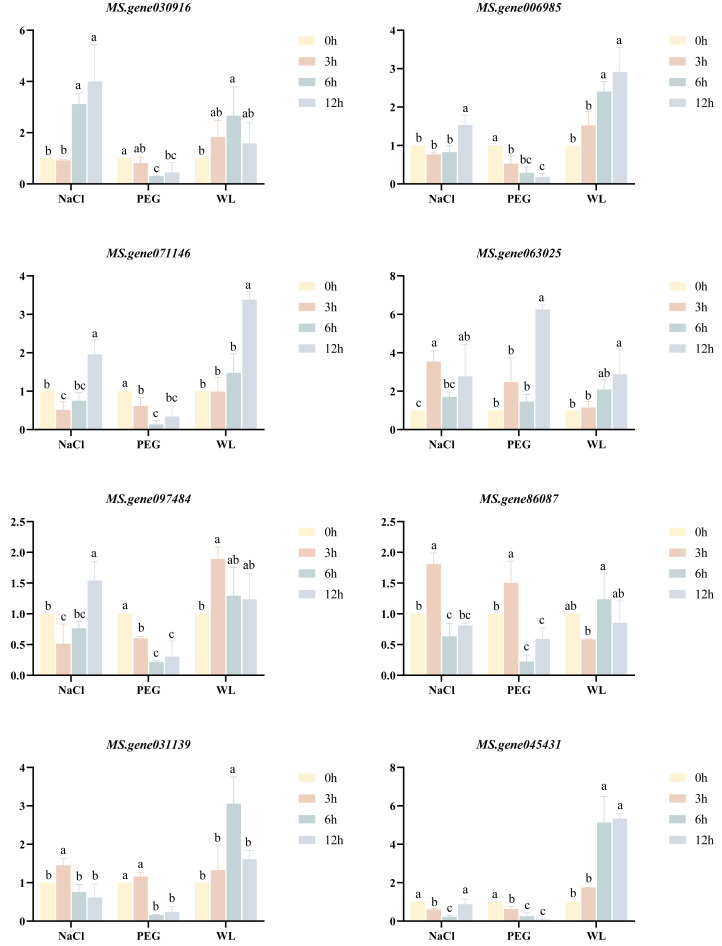
RT-qPCR expression analysis of eight *MsSUMO* genes under different abiotic stress conditions. Relative expression levels were measured at 0, 3, 6, and 12 h after NaCl, PEG, and waterlogging (WL) treatments using RT-qPCR. Data were normalized to the internal reference gene, and vertical bars indicate standard deviation.

To further validate the expression patterns of *MsSUMO* genes under salt (NaCl), drought (PEG), and waterlogging (WL) treatments at different time points (0 h, 3 h, 6 h, and 12 h), eight *MsSUMO* family genes (*MS.gene30916*, *MS.gene006985*, *MS.gene071146*, *MS.gene063025*, *MS.gene097484*, *MS.gene86087*, *MS.gene031139*, and *MS.gene045431*) were selected for RT-qPCR analysis (Fig. 8). The findings revealed unique expression profiles for each gene under various stress conditions: Under salt stress, the expression of *MS.gene30916* increased significantly over time. *MS.gene006985*, *MS.gene071146*, and *MS.gene097484* were slightly downregulated at 3–6 h, but reached their highest expression levels at 12 h. *MS.gene063025*, *MS.gene86087*, and *MS.gene031139* peaked at 3 h. *MS.gene045431* showed a pattern of initial downregulation followed by a slight increase, but overall remained downregulated. Under drought stress, *MS.gene006985* and *MS.gene045431* exhibited a time-dependent decrease in expression. *MS.gene063025* was upregulated, with its highest expression at 12 h. *MS.gene30916*, *MS.gene071146*, and *MS.gene097484* were downregulated, showing the lowest expression at 6 h. *MS.gene86087* and *MS.gene031139* were initially upregulated, peaking at 3 h, and then declined. Under waterlogging stress, *MS.gene30916* and *MS.gene031139* showed increased expression at 3 h and peaked at 6 h, followed by a decline. *MS.gene097484* was continuously upregulated, with the highest expression at 3 h. *MS.gene86087* was downregulated at 3 h and then significantly upregulated at 6 h. *MS.gene006985*, *MS.gene071146*, *MS.gene063025*, and *MS.gene045431* exhibited a time-dependent increase in expression, reaching their highest levels at 12 h.

**Figure 9:**
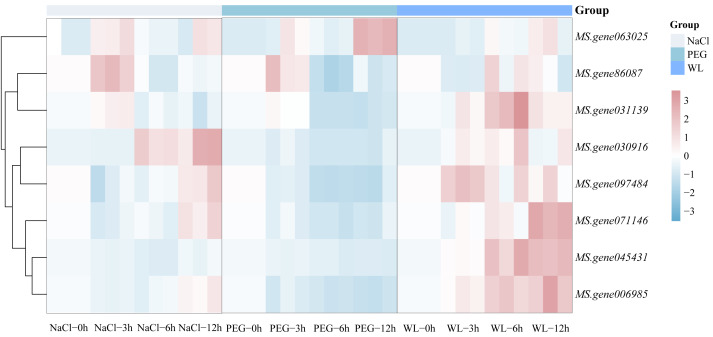
Clustering analysis of the expression trends of eight *MsSUMO* genes under abiotic stresses. Time points (0 h, 3 h, 6 h, 12 h) and conditions (NaCl, PEG, WL) were used for clustering. Genes with early induction or stress-specific patterns were highlighted.

Based on the clustering analysis of gene expression patterns (Fig. 9), *MS.gene063025*, *MS.gene86087*, and *MS.gene031139* showed significant upregulation at 3 h or 6 h under NaCl treatment, followed by a decline at 12 h, suggesting an early-induction expression pattern in response to salt stress. Under PEG-induced drought stress, the expression levels of most genes were downregulated, particularly *MS.gene063025* and *MS.gene86087*, indicating that these genes may be repressed under drought conditions. In contrast, under waterlogging (WL) stress, genes such as *MS.gene097484*, *MS.gene071146*, and *MS.gene006985* were significantly upregulated at 6 h and 12 h, suggesting that these genes might serve as positive regulators in the plant’s response to waterlogging stress.

## Discussion

Alfalfa plays a key role in sustainable livestock production and food security and is an important leguminous forage crop widely cultivated worldwide. However, in practical production, alfalfa is prone to various abiotic stresses, which can severely affect its yield and quality. *SUMO*ylation plays a vital role in plant responses to abiotic stresses. Although *SUMO* genes have been extensively studied in species such as *Arabidopsis* (Kurepa et al., 2003; Park et al., 2011), tomato (Alamery et al., 2020), potato (Ghimire et al., 2020), and soybean (Li et al., 2017), their functions in alfalfa remain largely unexplored. In this study, the *SUMO* gene family in alfalfa was analyzed using bioinformatics approaches, and 49 genes belonging to the *MsSUMO* family were identified. This number is greater than that observed in diploid species such as rice (Shimada & Tanaka, 2023) and maize (Augustine et al., 2016), likely due to the autotetraploid nature of alfalfa. Thus, the large number of *SUMO* genes in alfalfa is closely associated with its polyploidy. Subcellular localization analysis revealed that 45 *MsSUMO* proteins were localized in the cytoplasm, while 29 were present in both the nucleus and cytoplasm, consistent with the general localization patterns of *SUMO* proteins and their modification systems in other plant species (Ryu, 2022; Srivastava & Sadanandom, 2020). Phylogenetic analysis classified the *MsSUMO* genes into seven subfamilies (Groups I–VII). Among them, Groups II, III, and V contained members from all three species, and this cross-species distribution indicates that the *SUMO* gene family is highly conserved during evolution, suggesting its indispensable roles in fundamental physiological and metabolic processes in plants. In contrast, Groups IV, VI, and VII were composed almost exclusively of alfalfa members, which may be associated with whole-genome duplication events in alfalfa as an autotetraploid species. From the perspective of phylogenetic relationships, alfalfa showed a closer clustering with soybean, another legume species, across several branches. This pattern is consistent with plant evolutionary relationships and further suggests that *SUMO* genes in legume species share similar genetic characteristics.

The gene structures and conserved domains of each subfamily are highly similar. In the analysis of conserved motifs within the *MsSUMO* family, Motif 1 and Motif 2 were found to be present in all 31 SUMO proteins of alfalfa, while Motif 3 and Motif 6 appeared in 18 SUMO proteins, and 16 members simultaneously contained Motif 5 and Motif 7. These top 10 motifs, characterized by significant *E*-values and consistent distribution, effectively represent the core structural conservation and functional potential of the *MsSUMO* family. The identified *MsSUMO* genes exhibited a relatively simple structure, with 25 *MsSUMO* genes lacking introns. Members within the same subfamily shared similar or identical types and numbers of conserved motifs, whereas noticeable differences were observed among different subfamilies. Collinearity analysis of the *MsSUMO* gene family further revealed the presence of tandem duplication relationships among some genes.

Cis-acting element analysis of the *MsSUMO* gene family revealed the presence of elements such as ABRE, ARE, and TGACG-motif, which mediate responses to abiotic stresses. Notably, ABRE was present in the promoter regions of nearly all *MsSUMO* genes, suggesting its role in regulating the expression of *SUMO* genes in alfalfa under drought and salt stress. This is consistent with studies in *Arabidopsis*, where ABRE elements in the *DREB2A* promoter act as core switches for drought and high-salinity induced expression (Kim et al., 2011). In rice, MYC2-like transcription factors were found to positively regulate *OsCYP2* expression by directly binding to ABRE, thereby significantly enhancing salt tolerance (Liu et al., 2022). The presence of ARE elements in the promoters of *MsSUMO* genes suggests that the *SUMO* gene family may participate in the response to waterlogging stress through the ABA signaling pathway. In maize, deletion of three ARE elements completely abolished promoter activity, directly demonstrating that AREs are essential cis-acting elements for the induction of *zmzf* promoters under waterlogging conditions (Du, Zhang & Li, 2010).

The PPI network revealed the core mechanisms of the SUMOylation system in alfalfa. Among these components, G7IBK6 (UBC9/SCE1) directly determines the intensity of SUMOylation through its expression level. Under drought stress, it activates the CBF cold-response pathway by modifying ICE1, while simultaneously negatively regulating the abscisic acid (ABA) signaling pathway to balance growth and stress tolerance (Park, Kim & Yun, 2016). G7LI77 (SIZ1) facilitates the SUMOylation of transcription factors such as DREB and AREB through substrate-specific recognition, thereby enhancing plant tolerance to salt and osmotic stress (Kujawska & Król, 2025). G7JDW1, as a SUMOylation-related protein, may contribute to maintaining the homeostasis of the modification system under changing stress conditions (Ulrich, 2005). The interactions between these core proteins and various stress-responsive proteins indicate that *MsSUMO* members coordinate with the SUMOylation enzyme complex to regulate the stability and activity of downstream effectors, thereby enhancing stress resistance in alfalfa. These findings provide a theoretical basis for future experimental validation of specific *MsSUMO* interaction networks in alfalfa.

Through transcriptome data analysis under various abiotic stress conditions, several *MsSUMO* genes were found to be differentially expressed in response to salt, drought, and waterlogging stress. Among them, eight genes were responsive to all three types of stress. To validate these results, RT-qPCR experiments were conducted. Under salt stress, four genes—*Ms.gene045431*, *Ms.gene031139*, *Ms.gene030916*, and *Ms.gene063025*—showed expression patterns consistent with those observed in the transcriptome analysis. Additionally, *Ms.gene006985*, *Ms.gene071146*, and *Ms.gene097484* exhibited consistent expression trends at 12 h, while *Ms.gene086087* was consistent at 3 h.

Under drought stress, *Ms.gene045431* and *Ms.gene006985* were upregulated in the transcriptome data but downregulated according to RT-qPCR results, which may suggest the involvement of post-transcriptional regulation or differential sensitivity to the intensity or duration of stress. The remaining genes showed expression patterns consistent with transcriptome data. Under waterlogging stress, *Ms.gene086087* at 6 h displayed similar expression trends to transcriptome results, and the other genes also exhibited expression consistent with the transcriptome data. These findings indicate that different *MsSUMO* genes exhibit relatively stable expression responses under distinct abiotic stress conditions, actively participating in abiotic stress responses. The results provide a theoretical basis for further functional characterization and regulatory mechanism studies of the *SUMO* gene family in alfalfa.

## Supplemental Information

10.7717/peerj.21276/supp-1 Supplemental Information 1 RT-qPCR test verification data of MsSUMO gene expression at different periods (0h, 3h, 6h, 12h) under salt (NaCl), drought (PEG), and flood (WL) treatments

10.7717/peerj.21276/supp-2 Supplemental Information 2 Based on the results of the hmmsearch program and further screening using the Batch CD-Search tool on the NCBI website, sequences lacking the typical SUMO domain were excluded. A total of 49 *MsSUMO* genes were identified from the Alfalfa ge

10.7717/peerj.21276/supp-3 Supplemental Information 3 MIQE checklist

10.7717/peerj.21276/supp-4 Supplemental Information 4 The protein sequences

10.7717/peerj.21276/supp-5 Supplemental Information 5 Chromosome location

10.7717/peerj.21276/supp-6 Supplemental Information 6 Raw data

10.7717/peerj.21276/supp-7 Supplemental Information 7 The primers sequences of RT-qPCR

## Additional Information and Declarations

### Data Availability

The following information was supplied regarding data availability: The data are available in the Supplemental Files. The genome and proteome sequences of *Medicago sativa* (alfalfa) are available at AlfalfaGEDB. The complete lists of the 49 gene accessions are available in the Supplemental Files.
